# The Effectiveness of Patient Training in Inflammatory Bowel Disease Knowledge via Instagram: Randomized Controlled Trial

**DOI:** 10.2196/36767

**Published:** 2022-10-19

**Authors:** Dominik Blunck, Lena Kastner, Michael Nissen, Jacqueline Winkler

**Affiliations:** 1 Department of Health Management Institute of Management Friedrich-Alexander-Universität Erlangen-Nürnberg Nuremberg Germany; 2 Machine Learning and Data Analytics Lab Department Artificial Intelligence in Biomedical Engineering Friedrich-Alexander-Universität Erlangen-Nürnberg Erlangen Germany; 3 Bristol-Myers Squibb GmbH & Co KGaA Munich Germany

**Keywords:** social media, Instagram, patient training, patient education, disease-related knowledge, RCT, randomized controlled trial, Germany, inflammatory bowel disease, IBD-KNOW

## Abstract

**Background:**

Patients’ knowledge was found to be a key contributor to the success of therapy. Many efforts have been made to educate patients in their disease. However, research found that many patients still lack knowledge regarding their disease. Integrating patient education into social media platforms can bring materials closer to recipients.

**Objective:**

The aim of this study is to test the effectiveness of patient education via Instagram.

**Methods:**

A randomized controlled trial was conducted to test the effectiveness of patient education via Instagram among patients with inflammatory bowel disease. Participants were recruited online from the open Instagram page of a patient organization. The intervention group was educated via Instagram for 5 weeks by the research team; the control group did not receive any educational intervention. The knowledge about their disease was measured pre- and postintervention using the Inflammatory Bowel Disease Knowledge questionnaire. Data were analyzed by comparing mean knowledge scores and by regression analysis. The trial was purely web based.

**Results:**

In total, 49 participants filled out both questionnaires. The intervention group included 25 participants, and the control group included 24 participants. The preintervention knowledge level of the intervention group was reflected as a score of 18.67 out of 24 points; this improved by 3 points to 21.67 postintervention. The postintervention difference between the control and intervention groups was 3.59 points and was statistically significant (*t*_32.88_=–4.56, 95% CI 1.98-5.19; *P*<.001). Results of the regression analysis, accounting for preintervention knowledge and group heterogeneity, indicated an increase of 3.33 points that was explained by the intervention (*P*<.001).

**Conclusions:**

Patient education via Instagram is an effective way to increase disease-related knowledge. Future studies are needed to assess the effects in other conditions and to compare different means of patient education.

**Trial Registration:**

German Clinical Trials Register DRKS00022935; https://tinyurl.com/bed4bzvh

## Introduction

### Inflammatory Bowel Disease

Inflammatory bowel disease (IBD) is a group of chronic inflammatory diseases of the gastrointestinal tract. IBD can be divided into Crohn disease, ulcerative colitis, and other diseases that present with different gastrointestinal symptoms, such as diarrhea [1]. The global prevalence of IBD is approximately 3.9 million females and 3.0 million males, with a worldwide accelerating incidence [2,3]. The economic burden of IBD is highly relevant. Annual costs per patient were shown to be 3-fold in IBD patients compared to patients without IBD [4]. A systematic review estimated the mean annual health care cost of IBD patients in North America to be over US $13,000 [5]. Although the disease is not yet fully understood [6], there exist different pharmaceutical and nonpharmaceutical interventions. For pharmaceutical interventions, aminosalicylates, corticosteroids, antibiotics, immunomodulative treatments, and different biologic treatments are used, depending on the clinical stage of IBD [7-10]. Nonpharmaceutical interventions are surgery—for example, for patients who are refractory to treatment—and other interventions, such as diets [7]. Because of a greater likelihood of depression or anxiety, resulting in lower quality of life, psychotherapy is a common therapeutic approach as well [7,11-13].

Studies show that IBD patients benefit from higher disease-related knowledge, which has positive effects on the clinical outcomes of their overall therapy [14,15]. Not only in IBD, but also in other, especially chronic, conditions, higher levels of knowledge of the respective condition are related to better outcomes [16,17]. Besides the clinical importance, improving patients’ disease-related knowledge is also economically important. A study by Colombara et al [18] found that an increase of 5 points in patients’ disease-related knowledge on a 24-point scale could decrease costs in the first year after diagnosis by over €1000.

### Disease-Related Knowledge

In this section we describe (1) why higher disease-related knowledge might positively affect clinical outcomes, (2) how other studies approached increasing disease-related knowledge in IBD, (3) how we propose to integrate patient education into patients’ daily lives via social media, and (4) how others did so for other indications.

Higher disease-related knowledge has a positive effect on clinical outcomes because it improves adherence and enables shared decision-making, which ultimately leads to better clinical outcomes. Adherence to the treatment plan is a major success factor in therapy. However, in chronic diseases in particular, studies found that medication adherence often is insufficient [19,20]. Higher levels of patient knowledge showed improved adherence in different conditions, for example, because of higher motivation or dispelled misbeliefs [21,22]. Several studies found an improvement in adherence among patients with IBD through different educational interventions and, subsequently, higher rates of knowledge of IBD [23,24]. Bucci et al [25] investigated the factors that predict adherence among Italian patients with IBD and described the complex treatment plan for IBD, which requires taking different pharmaceuticals as well as lifestyle and nutrition changes. Hence, the literature implies a need to enhance knowledge of IBD and related therapies for better adherence. In one study by Elkjaer et al [26], patients with IBD who participated in dedicated educational programs showed better compliance and adherence, higher disease-related knowledge, better quality of life, and better coping with relapsing, leading to a mean relapse duration of 18 days compared to 77 days in the control group. Shared decision-making improves clinical outcomes because therapy plans are aligned with patients’ values, lifestyles, and expectations [27-29]. In IBD, shared decision-making is a relevant factor regarding medication therapy [30]. For shared decision-making, however, equitable collaboration between patients and physicians is required. Therefore, high levels of disease-related knowledge are necessary to enable a common understanding of the underlying problems and therapy options [29,31]. Additionally, the majority of patients with IBD also want to be actively involved in the decision-making process, as surveys have shown [32-34], which might be due to high levels of uncertainty associated with IBD [35]. Thus, one important antecedent of shared decision-making is informing patients.

In the case of IBD, different methods to increase disease-related knowledge have been studied. One study compared a telemedicine intervention (ie, SMS text messaging) with standard care (ie, educational materials at clinical appointments) to increase disease-related knowledge in IBD. On a 24-point scale, telemedicine increased the baseline value of 12.6 by 2.4 points, whereas standard care only yielded 1.8 points [36]. In a study where patients received a CD-ROM for self-paced autodidactic learning, participants were able to increase their knowledge from 12.2 points on a 30-point scale to 19.9 points, an increase of 7.6 points. After 9 months of follow-up, the knowledge increase was still 5.3 points higher than at baseline [37]. Another study compared a 12-hour structured education program with standard care (ie, teaching by physician during regular visits). On a 24-point scale, the intervention group’s disease-related knowledge increased by 7.71 points immediately after the intervention and 7.94 points after 8 weeks compared to baseline. The control group’s disease-related knowledge increased by 3.55 points immediately after standard care and 4.05 points after 8 weeks compared to baseline [38]. In another study, IBD patients were educated through counseling, pill cards, and educational material. In that study, knowledge increased from 8.15 points to 11.65 points [23].

Although different approaches for informing patients have already been studied, they might lack sustainable integration into patients’ daily lives. For example, Yin et al [39] argued that most of the educational apps they identified in a scoping review did not proactively inform patients, and patients instead had to access the app by themselves manually; this could be why they were poorly embedded into patients’ daily routines. In contrast, social media is discussed as a way to potentially overcome this problem, as many patients already use it and it comes with high interactivity [40].

Therefore, we suggest distributing information via Instagram. Instagram is a widely used social media platform with 1 billion users worldwide [41]. In most cases, Instagram is accessed via its corresponding smartphone app, which is used to view and share pictures or videos. Users can view pictures and videos in two ways: either via their timeline or the so-called *story* function. Media in the timeline is presented once to the user by the Instagram algorithm but is constantly available. Furthermore, the algorithm orders content as a result of user-based analyses. The story function is found in the top section of the Instagram home screen. Content creators can share short video clips or pictures in the story function, which are then presented to the creator’s followers. The order of the stories presented to a user also depends on user-based analyses. Instagram stories are available for 24 hours; however, creators can save their stories using the so-called “Story Highlights” feature, which makes stories constantly available. Buttons to view different categories of highlights are available on every user profile. Besides the sole presentation of pictures or videos in the story, creators can also integrate different interactive functionalities, such as quizzes. A recent study evaluated the use of social media platforms and showed that 59% of Instagram users visited Instagram at least daily, and more than one-third of the users visited the app several times a day [42]. Therefore, it seems like a reasonable approach for integrating patient education into everyday life.

Previous studies of social media–based interventions showed overall good results in improving clinical outcomes and patients’ disease-related knowledge about different conditions, for example, diabetes [43]. A review article by Grajales et al [44] reported various approaches for applying social media to health care and patient education. For example, several apps in Facebook are described as well as weblogs. Another paper studied the effect of participation in social health networks on patient activation. Patients with a chronic condition participated in a dedicated social network where they could find medical advice from experts as well as the opportunity to connect with other patients. Higher frequency and duration of usage of this network was associated with higher patient activation, and patients felt more empowered [45].

### Aim

The purpose of this study is, thus, to explore whether patient education via Instagram stories is an effective method for educating and informing adult patients with IBD, as compared to patients receiving no intervention, by conducting a randomized controlled trial (RCT).

## Methods

### Design

This study was conducted as a 2-arm, parallel-group, purely web-based RCT, following the CONSORT-EHEALTH (Consolidated Standards of Reporting Trials of Electronic and Mobile Health Applications and Online Telehealth) guideline [46]. The intervention group received disease-related education for 5 weeks, and the control group did not receive any educational treatment. Outcomes were assessed before and after the intervention.

### Recruitment and Randomization

For recruitment, we were supported by CHRONISCH GLÜCKLICH e.V., a German patient organization for IBD. The organization owns and operates an Instagram page that had 2332 followers (87.5% female) at the start of recruitment. Comparable pages have similar demographics. They announced the study in their publicly available “Instagram Stories” and called for participation. Participants were included if they met the following inclusion criteria: (1) were older than 18 years of age, (2) had an Instagram account, and (3) were able to fill out a questionnaire. After a recruitment period of 2 weeks, we assigned the participants to either the intervention group or the control group with the help of the online program Research Randomizer [47].

### Dropout Effects

According to the intention-to-treat concept, we included all data from all patients in our analysis, whether or not they followed the study protocol [48]. To ensure robustness of our results, we conducted all analyses without dropouts. To better understand dropout effects, we investigated group differences between included participants and those who dropped out with respect to current age, age at diagnosis, sex, diagnosis, and prestudy disease-related knowledge.

### Intervention

**Figure 1:**
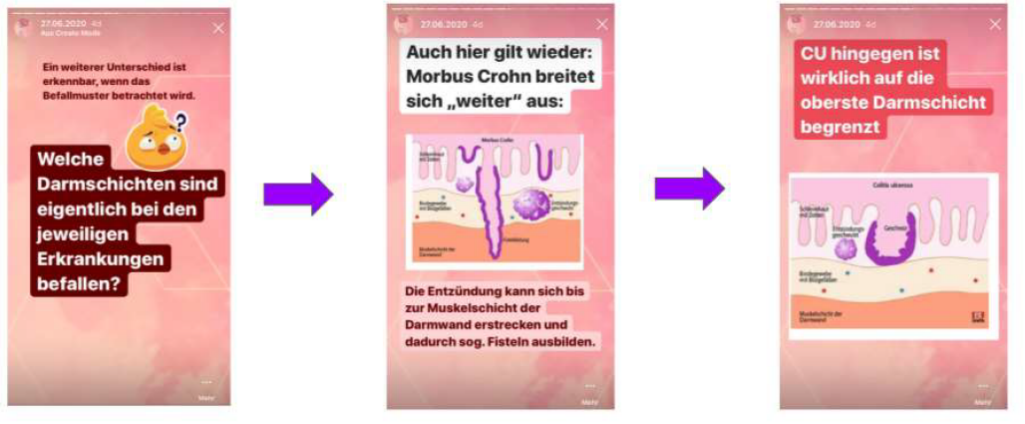
Example screenshots of educational material [content in German].

The intervention group received access to a nonpublic Instagram account, which posted educational material to the story function one to three times per week from June 29, 2020, to July 31, 2020. Furthermore, the stories were saved using the highlights function to be watched later. The posted educational material was either informational or interactive (Figure 1). All educational content was publicly available information about IBD and was reviewed by a physician before being posted by the research team. For interactive purposes, quizzes, for example, were included in the educational stories. Furthermore, participants were not forced or controlled to watch the Instagram stories; they solely received access and followed the account. If participants provided feedback or made requests during the study, such as comments on a story, this was incorporated into successive stories over the 5-week period (ie, higher contrast).

### Outcome Measure

The study’s primary outcome was patients’ knowledge about IBD. The outcome was measured at baseline (ie, preintervention) and 1 week after the last story was published (ie, postintervention). We measured patients’ knowledge by self-assessment using an online questionnaire. There exist different validated questionnaires to measure patients’ knowledge about IBD, such as the Crohn's and Colitis Knowledge score [49] and the Inflammatory Bowel Disease Knowledge (IBD-KNOW) questionnaire [50]. We chose the IBD-KNOW questionnaire because it is newer and includes a broader field of disease and therapy-related knowledge, such as biologics. We measured the patients’ knowledge about IBD by using the validated IBD-KNOW questionnaire. For this purpose, we translated the original English-language questionnaire into German (Multimedia Appendix 1). This translated version was reviewed by a physician. The questionnaire consists of 24 items, asking questions about IBD facts with response options of “true,” “false,” and “I don’t know.” The number of correct answers—“I don’t know” is not counted as correct—represents the respondent’s level of knowledge about IBD and, hence, the score ranges from 0 to 24 points. The online questionnaire was evaluated by application of the Checklist for Reporting Results of Internet E-Surveys (CHERRIES) [51].

Besides the 24 IBD-specific questions, we included several sociodemographic and disease-related variables in the questionnaire, which were included as control variables in the regression analyses.

### Sample Size

To identify the required sample size, we performed a power analysis. An improvement of 3 points in the IBD-KNOW score has been previously regarded as clinically important [36,52]. At an SD of 4.7 [15] and to detect group differences of at least 3 points on the IBD-KNOW scale, with power greater than 0.8 and α<.05, a sample size of 40 participants per group was required [53]. We anticipated a dropout rate of 20%, giving a total planned sample size of 100 participants.

### Statistical Analysis

#### Overview

We analyzed the study’s data in three ways. Firstly, we descriptively analyzed the study participants’ characteristics. Secondly, we conducted inferential statistics to display group and time differences in level of knowledge. Thirdly, we conducted a regression analysis. All statistical analyses were performed with R statistical software (version 4.0.0; R Foundation for Statistical Computing) [54,55]. We used the following R packages: pwr for power calculation [53], ggplot2 for data visualization [56], car for calculating variance inflation factors [57], and dplyr and tidyr for data management [58,59]. *P* values of less than .05 were considered statistically significant.

#### Inferential Statistics

To analyze group differences regarding categorical variables, we used the chi-square test. For continuous variables, we conducted the Welch *t* test.

#### Regression Analysis

To further analyze the effects, account for group heterogeneity, and ensure robustness of our results, we estimated an ordinary least squares (OLS) regression model of patients’ knowledge with a difference-in-differences approach (ie, lm()-function in R).

The dependent variable in the regression model was the IBD-KNOW score. The independent variables included a group dummy variable, a time dummy variable, and an interaction term of group and time. The group dummy value was 1 for the treatment group and 0 for the control group; the time dummy value was 1 for the postintervention questionnaire and 0 for the preintervention questionnaire. The covariates were chosen to control for further effects that are associated with learning. Hence, we controlled for sex (dummy variable, female = 1), age in years, the duration in years that the patient has lived with their IBD diagnosis at the time of the study (ie, current age – age at diagnosis), and diagnosis (dummy variable for Crohn disease) [60,61]. This is reflected in the following equation:

*y* = *β*_0_ + *β*_1_*dSex* + *β*_2_*dDiagnosis* + *β*_3_*Age* + *β*_4_*Duration* + *β*_5_*dTime* + *β*_6_*dGroup* + *β*_7_(*dTime* × *dGroup) + e*

In the regression analysis, we followed the intention-to-treat approach by including all dropouts in the analysis. However, we estimated further models with dropouts excluded to ensure robustness of the results. Multicollinearity was checked by calculating variance inflation factors. Values greater than 5 were considered to indicate multicollinearity [62].

### Ethics Approval

This study was prospectively approved by the Ethics Committee of Friedrich-Alexander-Universität Erlangen-Nürnberg (reference No. 202_20 B) and retrospectively registered in the German Clinical Trials Register (DRKS00022935). All participants declared informed consent before the study after receiving patient information and the data privacy declaration.

## Results

**Figure 2:**
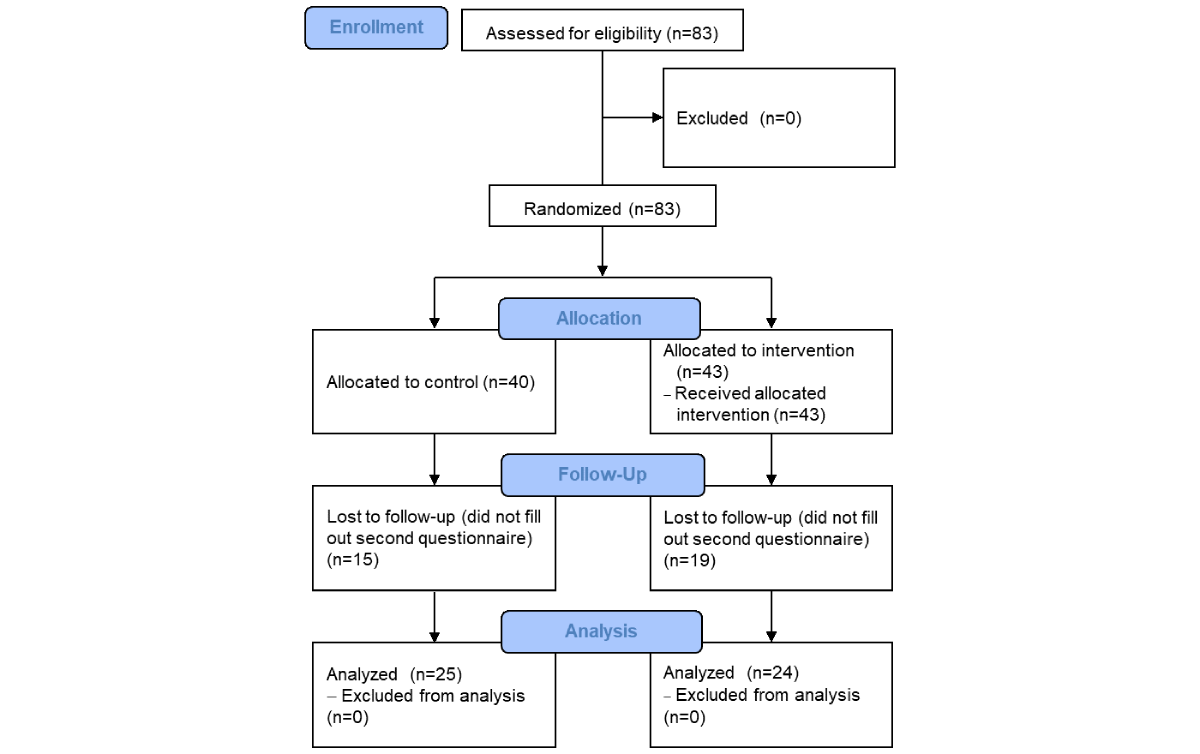
Flowchart of participants.

Out of 83 initial participants, 40 (48%) were assigned to the control group and 43 (52%) were assigned to the treatment group. In total, 15 participants from the control group and 19 from the intervention group were lost to follow-up because they did not fill out both questionnaires and were, thus, regarded as dropouts. This left a total of 49 participants—25 (51%) in the control group and 24 (49%) in the intervention group—who were analyzed (Figure 2). However, all outcome analyses are reported with and without dropouts in this section. The characteristics of the intervention and control group participants are displayed in Table 1; we did not find statistically significant differences between the control and intervention groups.

We did not find significant group differences between the included participants and the dropout group with respect to age at diagnosis (*P*=.34), sex (*P*=.37), type of diagnosis (*P*=.93), and prestudy IBD knowledge (*P*=.17). A difference in age between the dropout group and the included participants was found (*P*=.04), with the dropouts being 3 years older on average. This difference in age did not yield a difference regarding the length of IBD history, which is the difference between current age and age at diagnosis (*P*=.27).

**Figure 3:**
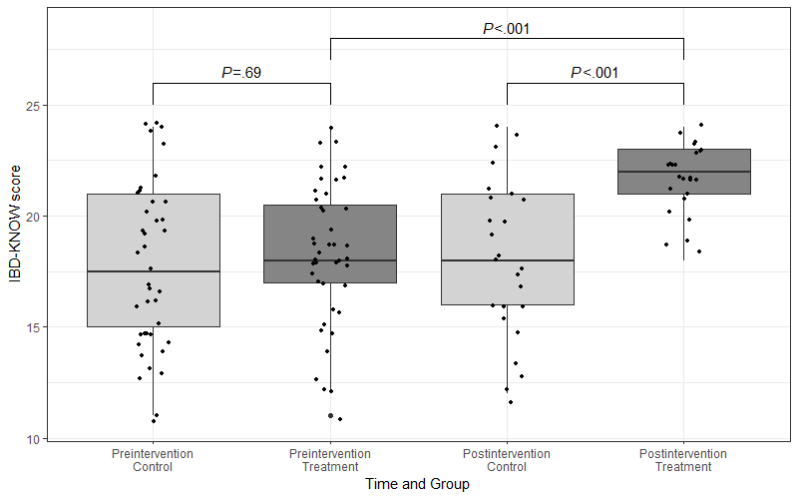
Levels of pre- and postintervention knowledge by control and intervention groups. IBD-KNOW: Inflammatory Bowel Disease Knowledge.

Without excluding dropouts (ie, intention-to-treat approach), preintervention knowledge in the control group was reflected by a mean of 17.73 (SD 3.72) points, and preintervention knowledge in the intervention group was reflected by a mean of 18.33 (SD 3.13) points; the difference was not statistically significant (*t*_76.47_=–0.79, 95% CI –2.11 to 0.91; *P*=.43). When dropouts were excluded, preintervention knowledge in the whole sample was reflected by a mean of 18.47 (SD 3.40) points. With dropouts excluded, preintervention knowledge in the control group was reflected by a mean of 18.28 (SD 3.76) points, and preintervention knowledge in the intervention group was reflected by a mean of 18.67 (SD 3.05) points. The difference between the control and intervention groups before the intervention was not statistically significant (*t*_45.73_=–0.40, 95% CI –2.35 to 1.58*; P*=.69). Postintervention knowledge was reflected by a mean of 18.08 (SD 3.60) points in the control group and 21.67 (SD 1.55) points in the intervention group. This difference of 3.59 points was statistically significant (*t*_32.88_=–4.56, 95% CI –5.19 to –1.98; power=0.99; *P*<.001). The pre- and postintervention knowledge levels by the control and intervention groups are displayed in Figure 3.

**Table 1:** Characteristics of the study participants.

Characteristics		Control group (n=25)	Intervention group (n=24)	Full sample (N=49)	*t* test^a^ (*df*)	*χ*^2a^ (*df*)	*P* value
Age (years), mean (SD)		25.88 (5.82)	26.96 (6.69)	26.41 (6.22)	–0.60 (45.53)	N/A^b^	.55
Age at diagnosis (years), mean (SD)		21.40 (6.42)	19.88 (8.07)	20.65 (7.24)	0.73 (43.91)	N/A	.47
**Sex, n (%)**							
	Female	23 (92)	24 (100)	47 (96)	N/A	0.5 (1)	.49
	Male	2 (8)	0 (0)	2 (4)	N/A	—^c^	—
**Type of diagnosis, n (%)**							
	Crohn disease	14 (56)	16 (67)	30 (61)	N/A	0.2 (1)	.64
	Ulcerative colitis	11 (44)	8 (33)	19 (39)	N/A	—	—
**Knowledge about IBD^d^, IBD-KNOW^e^ score, mean (SD)**							
	Preintervention	18.28 (3.76)	18.67 (3.05)	18.47 (3.40)	–0.40 (45.73)	N/A	.69
	Postintervention	18.08 (3.60)	21.67 (1.55)	19.84 (3.31)	–4.56 (32.88)	N/A	<.001

^a^The *t* test (2-tailed) and chi-square test were used to measure the difference between the control and intervention groups.

^b^N/A: not applicable; this test was not applied to this variable.

^c^The chi-square value and its related *P* value for a group are reported in the top row for that group.

^d^IBD: inflammatory bowel disease.

^e^IBD-KNOW: Inflammatory Bowel Disease Knowledge; scores range from 0 to 24 points.

The results of the OLS regression analysis are displayed in Table 2. Model 1 shows the baseline effect of the selected control variables on patients’ knowledge scores. Model 2 adds the time and group dummy variables, as well as the interaction term for these variables. The variable of interest is the interaction term, as it describes the main treatment effect. Patients in the treatment group increased their knowledge score by 3.07 points, all other things being equal, compared to the control group (*P*=.001; see Multimedia Appendix 2 for a visualization of the treatment effect).

*R*^2^ represents the proportion of variance in the dependent variable that is explained by the model. In model 1, 1% of the variance in patients’ knowledge is explained by the control variables. After adding the independent variables, the *R*^2^ of model 2 shows that 18% of the variance of patients’ knowledge is explained by the variables. The adjusted *R*^2^, which considers the number of control variables, in model 2 indicates that 13% of the variance is explained by model 2, a gain of 15 percentage points (pp) compared to model 1. The statistically significant *F* test values in model 2 indicate an overall significant model [61].

Variance inflation factors were all well below the cutoff of 5, with a maximum in model 1 of 1.06 in age and a maximum in model 2 of 2.57 in the interaction term; this was expected, as the interaction was a linear combination of two other variables. Given these results, we do not consider multicollinearity to be a major problem in our analysis.

Results of the robustness test, including the participants who dropped out, confirmed our results: estimate of time × treatment = 3.21 (*P*=.01); adjusted *R*^2^=0.17; *F*_7,90_=3.83 (*P*=.001). The results can be found in Table S1 in Multimedia Appendix 3.

Qualitative feedback from participants was incorporated during the study. For example, participants noted that some story slides were difficult to read, as IBD can affect patients’ eyes. Therefore, story slides were designed in high contrast after this feedback. Furthermore, we received a lot of positive feedback. Participants regarded the interventions as useful and meaningful. They also noted that they learned a lot—especially newly diagnosed participants—and stated that these interventions should be much more common.

**Table 2:** Difference-in-differences regression of knowledge about inflammatory bowel disease.

Variables and measures		Model 1			Model 2	
		Value	*P* value	Value		*P* value
**Control variables, estimated β coefficient (SE)**						
	Constant	19.82 (1.87)	<.001	19.72 (1.77)		<.001
	Female	–0.68 (1.29)	.60	–1.45 (1.19)		.23
	Crohn disease^a^	0.34 (0.63)	.59	0.16 (0.59)		.79
	Ulcerative colitis^a^	Reference	—^b^	Reference		—
	Age	–0.03 (0.05)	.49	–0.03 (0.05)		.53
	Duration	0.04 (0.06)	.51	0.00 (0.05)		.95
**Independent variables, estimated β coefficient (SE)**						
	Time^a^	N/A^c^	N/A	0.33 (0.83)		.69
	Intervention^a^	N/A	N/A	0.64 (0.72)		.38
	Time × intervention	N/A	N/A	3.07 (1.17)		.001
Observations, n		132	—	132		—
*R* ^2^		0.01	—	0.18		—
Delta *R*^2^		N/A	N/A	0.17		—
Adjusted *R*^2^		–0.02	—	0.13		—
Delta adjusted *R*^2^		N/A	N/A	0.15		—
*F* test (*df*)		0.320 (4, 127)	.86	3.889 (7, 124)		<.001

^a^Dummy variable.

^b^Not calculated.

^c^N/A: not applicable; model 1 was not applied to these variables or measures.

## Discussion

### Principal Findings

To answer the research question of whether educating adult patients with IBD via Instagram is effective, we conducted an RCT in a sample of 49 participants. After 5 weeks of training via Instagram stories, the intervention group yielded statistically significant and relatively higher levels of disease-related knowledge. Therefore, this study provides evidence for the effectiveness of patient education via Instagram.

With a mean of 76.95% correct answers (mean score of 18.47 out of 24), our sample showed an already-high mean knowledge level at baseline, compared to other studies in this area. For example, Abutaleb et al [36] found 52.50% correct answers during the preintervention stage. Others found mean baseline knowledge levels of 26.67% (8/30) [63], 33.33% (8/24) [18], 40.67% (12.2/30) [37], 40.79% (9.79/24) and 48.25% (11.58/24) [38], and 62.90% (18.87/30) [64]. Along with the relatively high baseline knowledge level, our study showed an increase in mean disease-related knowledge by 12.50 pp. Other studies achieved increases of 10 pp with telemedicine and 7.5 pp with standard interventions [36], 25.33 pp with a CD-ROM program [37], and 32.13 pp with a formal education program and 14.79 pp with a standard intervention [38]. Hence, the knowledge increase presented in our study is on the lower bound compared to other interventions. However, the study designs are not comparable without restrictions, for example, because of different intensity and frequency of interventions. Furthermore, higher baseline values come with less improvement from educational interventions [36], which is reasonable due to a saturation effect and a natural upper limit of the knowledge scale.

The dropout rate in this study was 41% (34/83) and was, thus, relatively high compared to other studies; for example, one study found 25% loss to follow-up after 6 months and 26% loss to follow-up after 12 months [36], whereas another study found 16% dropout immediately after the intervention and 22% loss to follow-up after 8 weeks [38]. We believe that the high dropout rate in our study may be due to the fact that, in order to prevent forced results, we did not send reminders to the participants to complete the questionnaires. Although the dropout group did not differ from the included participants regarding parameters such as length of IBD history or prestudy knowledge, dropouts were significantly older than included participants. A reason for this observation might be that older patients might have lower computer literacy and, thus, were more likely to drop out. Hence, future studies could address this issue in further elaborating the interplay of age and learning via social media in patients with IBD.

The unexpectedly high dropout rate ultimately led to a relatively low number of participants. This was not in line with the assumptions used for the power analysis. Future studies should take measures to either (1) expect a higher dropout rate and recruit a larger number of participants or (2) decrease the overall dropout rate. The latter may be achieved by using reminders or incentives. We did not take these measures in our study in order to reduce bias.

Finally, we found a high proportion of women among the followers of the organization specific to patients with IBD on Instagram. This may suggest that men generally have different coping strategies for dealing with IBD than women.

### Contribution

To our knowledge, this was the first study to analyze the effect of patient training via Instagram on patients’ disease-related knowledge. One main contribution of our study is evidence for the effectiveness of patient education via Instagram. Future work in this area should focus on disseminating educational content in regular care. One major challenge for this could be quality assurance because everybody could publish apparent educational content without expert review. If health care providers actively use social media platforms in the future, a high level of quality in educational material could be ensured. Another challenge might be the long-term motivation of users. Potential ways to reduce retention issues are high-quality content, high levels of monitoring and interaction, or the use of Instagram ads to increase visibility. However, the latter mechanism, in particular, might bias results in the study setting and would be more suitable in a regular care setting.

A difference in this study compared to previous studies is that participants in this study did not participate in dedicated trainings. This means that patients only received access to the Instagram account and were responsible for watching or actively participating. In classical patient educational interventions [38], patients actively participate in a training session, a physician visit, or similar. As it is not feasible in a regular care setting to ensure continued training via dedicated trainings, we contributed by providing a solution that is integrated into patients’ daily routines, without a cost to health care providers, and that can be used on a long-term and continued basis. Once educational material is designed and conceptualized, it could be used and reused in a large patient population. Compared to other, previously mentioned, ways of increasing patients’ disease-related knowledge, our approach is easy to implement, comes with good scalability, integrates educational content into patients’ lives, and addresses young people in particular. Furthermore, the proposed approach allows possibilities for patient organizations to closer engage with patients. Another application of educational social media interventions is the education of patients’ friends and family members. As those people are often affected or involved in the care of patients with chronic conditions, higher disease-related knowledge among friends and family members could also increase their understanding of patients’ situations and therapies, which subsequently would support patients. Furthermore, we contributed by providing a German translation of the IBD-KNOW questionnaire.

### Limitations

Our study comes with several limitations. First, patient recruitment took place via the Instagram pages of a German patient organization. This might bias and underestimate results for the total relevant population because we assumed that the patient organization’s Instagram page was being followed by an already-interested audience. For example, studies found that patients who are members of a patient organizations yield higher knowledge scores than patients who are not [50]. Therefore, the knowledge levels of this respective sample might already be above average. On the other hand, however, one could argue that the sample of patients could be more highly motivated and have a higher willingness to learn due to their higher level of interest, which counteracts this effect. Additionally, the study setting may have led to another selection bias because young and computer-literate people, in particular, are Instagram users, which limits generalizability. Another limitation might arise from dropouts. As 34 participants were lost to follow-up, our overall findings might be biased if the dropout probability was associated with the knowledge score, specifically with learning. Due to the unexpectedly high dropout rate, the sample size of our study was relatively small. Inclusion of larger study populations might be beneficial in gaining a better understanding of our findings.

Additionally, participants in this study were almost exclusively female. As the proportion of women among all patients with IBD is much lower [2], the generalizability of this study to the whole IBD population may be limited. However, the high proportion of women in our study is due to the demographic composition of Instagram followers of the patient organization with which we collaborated for recruitment.

To assert the sustainability of the effect of education via Instagram, further studies with a longer follow-up period are needed. However, the real-life setting of the proposed educational mode has a continuous character. This means that patients have continuous access to the educational material instead, for example, of a one-time visit at a seminar, which rather reduces the need for follow-up studies. Furthermore, previous studies found that the knowledge increase gained by patients with IBD stays relatively constant over time [37,38].

Additionally, we only considered German patients, which might reduce the generalizability of our results. Studies show that knowledge levels differ between countries [65]. Future studies should, therefore, focus on multicenter study designs or evaluate results across countries.

The interest in the educational material in our study might be higher than in a real-life setting because of a trial effect. Patients might be interested more or might learn more because they know they are part of a study [66] and not blinded. Therefore, the effect might be overestimated. To validate the effectiveness of patient education via Instagram or other social media channels, further research (eg, observational studies) is needed.

### Future Research

This study recommends different questions for future research. First, patient education via Instagram or other social media should be directly compared with other means of patient education, in order to compare effectiveness in a head-to-head comparison. Second, the effectiveness of Instagram patient education should be tested in other chronic conditions as well. Third, the economic effects of patient education via Instagram—or social media in general—should be explored. Integration into patients’ daily routines might reduce costs for transportation to a training facility or physician. Additionally, patient education via social media, such as Instagram, is easy to scale and increases accessibility, which leads to lower costs at training facilities or for physicians. Fourth, before rolling out Instagram patient education in regular settings, quality requirements should be defined to enable systematic dissemination and prevent communication of misleading or false information to patients.

### Conclusions

To test the effectiveness of patient training via Instagram, we conducted an RCT with 49 patients with IBD. The intervention group received access to an Instagram account, which posted educational material over 5 weeks. The outcome—patients’ knowledge about IBD—was measured at the pre- and postintervention stages using a questionnaire whose response scores ranged from 0 to 24 points. The intervention group yielded 3.59 more points than the control group, on average, after the intervention (*P*<.001), with no significant differences before the intervention. Therefore, we conclude that Instagram is an effective tool for educating patients and demonstrates large potential for future support of chronic conditions.

## Appendix

Multimedia Appendix 1 Inflammatory Bowel Disease Knowledge questionnaire.

Multimedia Appendix 2 Visualization of the treatment effect.

Multimedia Appendix 3 Difference-in-differences regression results (robustness check).

Multimedia Appendix 4 CONSORT-eHEALTH checklist (V 1.6.1).

## Abbreviations

CHERRIES: Checklist for Reporting Results of Internet E-Surveys
CONSORT-EHEALTH: Consolidated Standards of Reporting Trials of Electronic and Mobile Health Applications and Online Telehealth
IBD: inflammatory bowel disease
IBD-KNOW: Inflammatory Bowel Disease Knowledge
OLS: ordinary least squares
pp: percentage points
RCT: randomized controlled trial
