# Supplementary material for: The Effectiveness of Patient Training in Inflammatory Bowel Disease Knowledge via Instagram: Randomized Controlled Trial
Source: J Med Internet Res. 2022 Oct 19;24(10):e36767. doi: 10.2196/36767 (PMC9631171; doi:10.2196/36767)
Supplement: Multimedia Appendix 1 [file jmir_v24i10e36767_app1.docx]

# Multimedia Appendix 1: Questionnaire

## English translation of socio-demographic questions

1. Please provide your Instagram user name
2. Please provide your gender
   1. Male
   2. Female
   3. N/A
3. Please provide your diagnosis
   1. Crohn’s disease
   2. Colitis ulcerosa
   3. Other inflammatory bowel disease
   4. Unknown
   5. No inflammatory bowel disease
4. Please provide your age
5. What was your age at your first diagnosis?

## German version of IBD-KNOW Questionnaire

1. Das terminale Ileum ist der letzte Teil des Dünndarms. Es befindet sich im rechten Unterbauch.
2. Das Rektum ist ein Teil des Dickdarms. Es beginnt etwa 15 cm vom Anus entfernt und endet am Anus.
3. Die Hauptfunktion des Dickdarms ist die Aufnahme von Nährstoffen.
4. Menschen können ohne den Dickdarm, aber nicht ohne den Dünndarm überleben.
5. Es ist weitreichend bekannt, welche Lebensmittel bei chronisch-entzündlichen Darmerkrankungen vermieden werden sollen.
6. Die Raucherentwöhnung ist wichtig, um eine Verschlimmerung des Morbus Crohn zu verhindern.
7. Bei familiärer Vorbelastung ist das Risiko erhöht, ebenfalls eine chronisch-entzündliche Darmerkrankung zu entwickeln.
8. Eine chronisch-entzündliche Darmerkrankung kann sich in jeder Altersstufe entwickeln, tritt aber in jüngeren Jahren häufiger auf.
9. Bei anhaltend schwerer Entzündung kann sich eine Anämie entwickeln.
10. Morbus Crohn kann an jeder Stelle des Verdauungstraktes auftreten, vom Mund bis zum Anus.
11. Bei einer Colitis ulcerosa ist der Enddarm nur selten betroffen.
12. Entzündliche Darmerkrankungen können auch andere Organe als den Darm betreffen.
13. Eine chronisch-entzündliche Darmerkrankung gilt als geheilt, wenn Betroffene über mehrere Jahre hinweg symptomfrei sind.
14. Eine Entzündung im Darm kann weiterhin existieren, auch wenn eine Besserung der Symptome durch eine Therapie eintritt.
15. Eine langfristige Verabreichung von Steroiden wird empfohlen, um das Wiederauftreten von Entzündungen zu reduzieren.
16. Eine regelmäßige Entnahme von Blut mit anschließender Laboruntersuchung ist bei Patienten angezeigt, die immunsuppressive Substanzen wie Azathioprin einnehmen, da die Anzahl der weißen Blutkörperchen abnehmen kann.
17. Biologica werden hauptsächlich bei Patienten mit leichten Symptomen eingesetzt.
18. Zäpfchen oder Einläufe werden zur Behandlung von Blinddarmentzündungen bei Patienten mit Colitis ulcerosa verwendet.
19. Patienten, die seit 8-10 Jahren an einer chronisch-entzündlichen Darmerkrankung leiden, sollten sich einer Darmkrebs-Vorsorgeuntersuchung unterziehen.
20. Wenn eine Operation bei Colitis ulcerosa indiziert ist, wird immer eine permanente Kolostomie durchgeführt.
21. Bei Morbus Crohn mit Entzündung des Dünndarms kann die betroffene Person nach einer Operation geheilt sein.
22. Patienten mit chronisch-entzündlichen Darmerkrankungen sollten alle Medikamente absetzen, wenn eine Schwangerschaft in Erwägung gezogen wird.
23. Statt einer normalen Geburt wird dem Großteil der Frauen mit chronisch-entzündlichen Darmerkrankungen ein Kaiserschnitt empfohlen.
24. Immungeschwächte Menschen mit einer chronisch-entzündlichen Darmerkrankungen sollten jegliche Art von Impfung vermeiden.
