# Supplementary figures and images for: The Effectiveness of Patient Training in Inflammatory Bowel Disease Knowledge via Instagram: Randomized Controlled Trial
Source: J Med Internet Res. 2022 Oct 19;24(10):e36767. doi: 10.2196/36767 (PMC9631171; doi:10.2196/36767)

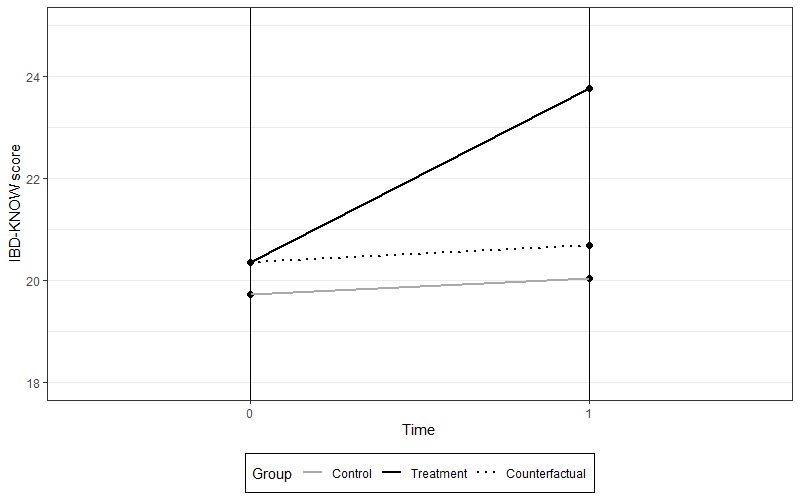

Supplement: Multimedia Appendix 2 [file jmir_v24i10e36767_app2.png]
