# Supplementary material for: The Effectiveness of Patient Training in Inflammatory Bowel Disease Knowledge via Instagram: Randomized Controlled Trial
Source: J Med Internet Res. 2022 Oct 19;24(10):e36767. doi: 10.2196/36767 (PMC9631171; doi:10.2196/36767)
Supplement: Multimedia Appendix 3 [file jmir_v24i10e36767_app3.docx]

**Multimedia Appendix 3**

Table S1: Difference-in-difference regression of knowledge on IBD (drop outs excluded)

| Variables and measures | | Model 1 | | | Model 2 | |
| --- | --- | --- | --- | --- | --- | --- |
|  | | Value | *P* value | Value | | *P* value |
|  | | | | | | |
| **Control variables, estimate (SE)** | | | | | | |
|  | Constant | 21.97 (2.44) | <.000 | 23.12 (2.26) | | <.001 |
|  | Female | –1.97 (1.78) | .273 | –3.40 (1.65) | | .043 |
|  | Crohn disease^a^ | 0.01 (0.73) | .988 | –0.41 (0.66) | | .535 |
|  | Ulcerative colitis^a^ | Reference | —^b^ | Reference | | — |
|  | Age | –0.04 (0.06) | .487 | –0.05 (0.05) | | .348 |
|  | Duration | 0.03 (0.07) | .723 | –0.04 (0.07) | | .527 |
| **Independent variables, estimate (SE)** | | | | | | |
|  | Time^a^ | N/A^c^ | N/A | –0.19 (0.88) | | .831 |
|  | Intervention^a^ | N/A | N/A | 0.87 (0.92) | | .349 |
|  | Time^a^ × intervention^a^ | N/A | N/A | 3.21 (1.25) | | .012 |
| Observations, n | | 98 | — | 98 | | — |
| *R*^2^ | | 0.02 | — | 0.23 | | — |
| Delta *R*^2^ | | N/A | N/A | 0.21 | | — |
| Adjusted *R*^2^ | | –0.02 | — | 0.17 | | — |
| Delta adjusted R^2^ | | N/A | N/A | 0.19 | | — |
| *F* statistic | | 0.43 | .786 | 3.83 | | .001 |

^a^Dummy variable.

^b^Not calculated.

^c^N/A: not applicable; model 1 was not applied to these variables or measures.
